# A new severity classification of lower limb secondary lymphedema based on lymphatic pathway defects in an indocyanine green fluorescent lymphography study

**DOI:** 10.1038/s41598-021-03637-6

**Published:** 2022-01-10

**Authors:** Akira Shinaoka, Kazuyo Kamiyama, Kiyoshi Yamada, Yoshihiro Kimata

**Affiliations:** 1 Department of Plastic and Reconstructive Surgery, Okayama University Graduate School of Medicine, Dentistry and Pharmaceutical Science, 2-5-1 Shikata-cho, Kita-ku, Okayama, 700-8558 Japan; 2 Department of Nursing, Kagawa Prefectural Central Hospital, Takamatsu, Japan

**Keywords:** Gynaecological cancer, Surgical oncology

## Abstract

Most protocols for lymphatic imaging of the lower limb conventionally inject tracer materials only into the interdigital space; however, recent studies indicate that there are four independent lymphatic vessel groups (anteromedial, anterolateral, posteromedial, and posterolateral) in the lower limb. Thus, three additional injection sites are needed for lymphatic imaging of the entire lower limb. We aimed to validate a multiple injection designed protocol and demonstrate its clinical benefits. Overall, 206 lower limbs undergoing indocyanine green fluorescent lymphography with the new injection protocol were registered retrospectively. To assess the influence of predictor variables on the degree of severity, multivariable logistic regression models were used with individual known risk factors. Using a generalized linear model, the area under the curve (AUC) of the conventional clinical model, comprising known severity risk factors, was compared with that of the modified model that included defects in the posterolateral and posteromedial groups. Multivariable logistic regression models showed a significant difference for the posteromedial and posterolateral groups. The AUC of the modified model was significantly improved compared to that of the conventional clinical model. Finding defects in the posteromedial and posterolateral groups is a significant criterion for judging lymphedema severity and introducing a new lymphedema severity classification.

## Introduction

Lymphedema is the chronic swelling of limbs caused by lymph stasis. Instances of secondary lymphedema related to cancer treatment, such as lymph node dissection, radiotherapy, and chemotherapy, are increasing steadily with the decrease in cancer deaths. Lymphedema does not merely cause hypokinesia but also immune dysfunctions, such as cellulitis, and reduces the quality of life and is a major concern for cancer survivors.

Previous studies have reported that 7‒77% patients post-breast cancer surgery^1–3^ and 0‒70% patients post-gynecological surgery^4–6^ developed lymphedema; radiotherapy^6–8^ or taxane-based chemotherapy^9,10^ was added to the risk of lymphedema onset. Moreover, considering patient characteristics, obesity and aging were recognized as risk factors of severity, in addition to risk factors for the onset of lymphedema^11–13^.

Considering the treatments for lymphedema, during the last half-century, decongestive lymphatic therapies, such as manual lymphatic drainage, elastic garments, and exercise, have been developed^14^. During the past two decades, plastic surgeons have attempted to improve the lymphatic function by lymphatic surgeries, such as lymphaticovenous anastomosis (LVA) and lymph node transfer (LNT)^15^. For an accurate therapeutic regimen, both decongestive therapy and surgery require information on the normal lymphatic pathways and pathological changes for each patient.

Lymphatic imaging can provide accurate information in this respect. Lymphoscintigraphy is the gold standard for lymphatic imaging^16^. Recently, new indirect imaging techniques, such as infrared fluorescent lymphography^17^, interstitial computed tomography-lymphography^18^, magnetic resonance imaging lymphography^19,20^, Single Photon-Emission Computed Tomography (SPECT), Computed Tomography (CT), Lymphoscintigraphy^21^, and photoacoustic lymphangiography^22^, have been developed. These methods permit the diagnosis of lymphedema by detecting micro-anatomical changes caused by lymphatic back-flow to the dermis (dermal back-flow, DB) and the reduced accumulation of tracer at the lymph node, concurrently yielding anatomical information regarding the lymphatic flow for treatments. However, these methods have a technical problem that they are based on the anatomical feature of the lymphatics. The lymphatics originate from numerous blind ends and only lymphatic capillaries surrounding the injection site can absorb the imaging materials. Moreover, currently, no comprehensive information exists regarding the lymphatic structures in the limbs in terms of the relationship among the capillaries, collecting vessels, and lymph nodes^23^.

**Figure 1:**
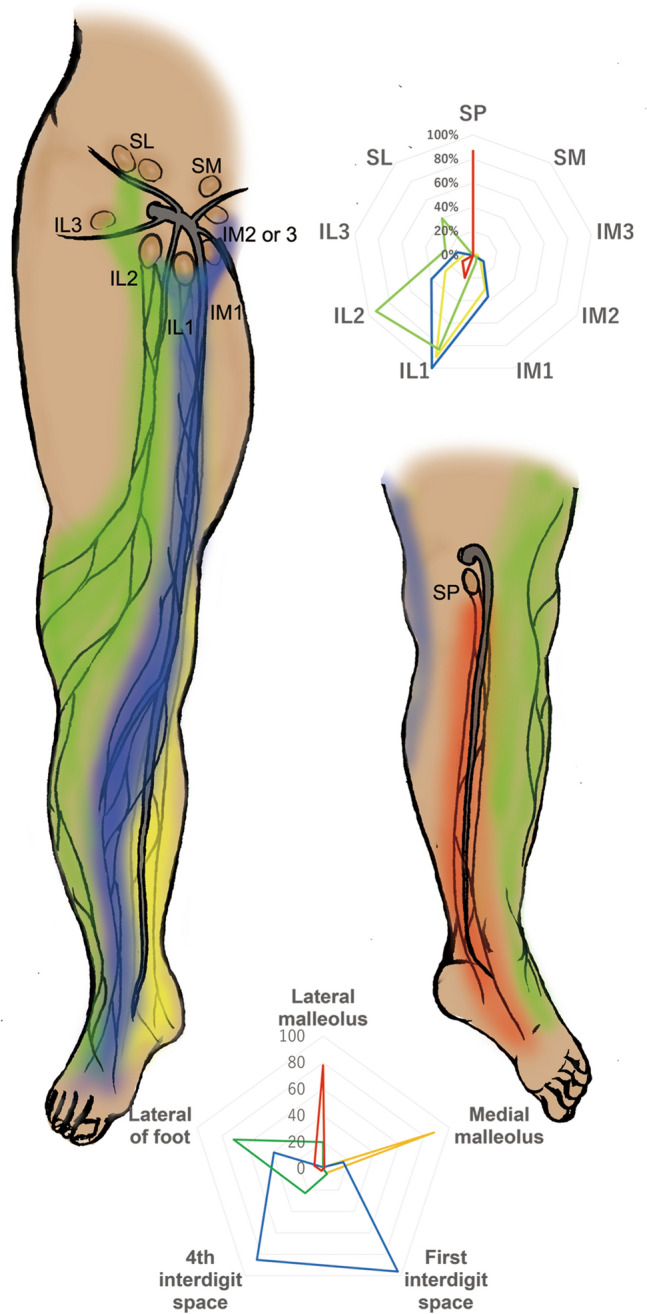
The relation between the four lymphatic vessel groups and lymph nodes in the lower limb. Lymphatic vessels in the lower limb were classified into four lymphatic vessel groups: anteromedial (blue), anterolateral (green), posteromedial (yellow), and posterolateral (red). These groups connect with primarily three lymph nodes: one superficial popliteal and two superficial inguinal lymph nodes (as detailed previously). The relationship between the injection sites and lymphatic vessel groups is shown in the lower radar chart. The number of axes show the percentage of the visualization of each lymphatic pathway. The color of the radar line denotes the four lymphatic pathways: anteromedial (blue), anterolateral (green), posteromedial (yellow), and posterolateral (red). Moreover, the relationship between lymphatic vessel groups and lymph nodes is shown in the upper radar chart. The number of axes show the percentage of the visualization of each lymph node. The color of the radar line denotes same lymphatic pathways in lower radar chart. *IL* inferior lateral, *IM* inferior medial, *SL* superior lateral, *SM* superior medial, *SP* superficial popliteal. This schema was drawn by A.S. with Adobe Photoshop CS6.13.6.2 (Adobe Inc., CA).

Most previous injection protocols of lymphatic imaging involved a single injection at the first interdigital space, because a single lymphatic network was presumably considered in the lower limbs and the interdigital spaces were sufficient to demonstrate the lymphatics related to the lower limbs as a whole. However, we previously investigated the details of the lymphatic anatomy of the lower limbs using a new lymphatic imaging technique in cadavers^24^ and demonstrated that the lymphatic vessels of the lower limbs could be classified into four independent groups^25^, and although there are many lymph nodes in the groin, only two lymph nodes in the lower side of the groin and a single popliteal lymph node correspond to the lower limbs (Fig. 1)^26^.

Based on this anatomical information, we established a new injection protocol to visualize the four independent lymphatic groups as a whole and interpret the lymphatic anatomical changes in the imaging examinations. The present study retrospectively investigated patients and control individuals who were examined using indocyanine green (ICG) fluorescent lymphography by our new injection protocol. Our aim was to verify this injection method for lymphography, to elucidate the importance of each lymphatic pathway, and to confirm the clinical benefits derived from detecting the defects in each of the lymphatic groups using this new injection technique.

## Results

According to ISL (International Society of lymphology) stage, the 164 lower limbs in the lymphedema group were divided into 46 stage 0 limbs, 35 stage 1 limbs, 58 stage 2 early limbs, 18 stage 2 delay limbs, and 7 stage 3 limbs. Compared to the control group, only the BMI (*p* = 0.0208) was statistically significantly different in the lymphedema group, while other characteristics showed no significant differences (Table 1).

**Table 1:** Characteristics of patients in control, in lymphedema, in mild type, and in sever type.

	All (n = 206)	Control group (n = 42)	Lymphedema group (n = 164)	P-value	Mild lymphedema (n = 81)		Sever lymphedema (n = 83)			P-value
					Stage 0 (n = 46)	Stage 1 (n = 35)	Stage 2e (n = 58)	Stage 2d (n = 18)	Stage 3 (n = 7)	
**Clinical characteristics**										
Age over 80 years, n (%)	14 (6.8)	0 (0)	14 (8.5)	0.0783	2 (4.3)	0 (0)	7 (12.1)	3 (16.7)	2 (28.6)	0.0096
Female sex, n (%)	196 (95)	42 (100)	154 (93.9)	0.219	44 (95.7)	35 (100)	52 (89.7)	16 (88.9)	7 (100)	0.0989
Body mass index kg/m^2^ over 25, n (%)	45 (21.8)	15 (35.7)	30 (18.3)	0.0208	5 (10.9)	9 (25.7)	9 (15.5)	5 (27.8)	2 (28.6)	0.841
Right, n (%)	103 (50)	19 (45)	83 (50.6)	0.605	28 (60.9)	19 (54.3)	26 (44.8)	8 (44.4)	2 (28.6)	0.0636
**Invasions for lymphatics**										
Lymph node dissection +, n (%)	141 (68)	0 (0)	141 (86)		38 (82.6)	31 (88.6)	50 (86.2)	16 (88.9)	6 (85.7)	0.825
Chemotherapy +, n (%)	96 (47)	0 (0)	96 (58.5)		30 (65.2)	22 (62.9)	34 (58.6)	7 (38.9)	3 (42.9)	0.157
Radiotherapy +, n (%)	43 (21)	0 (0)	43 (26.2)		8 (17.4)	8 (22.9)	19 (32.8)	5 (27.8)	3 (42.9)	0.0764

**Figure 2:**
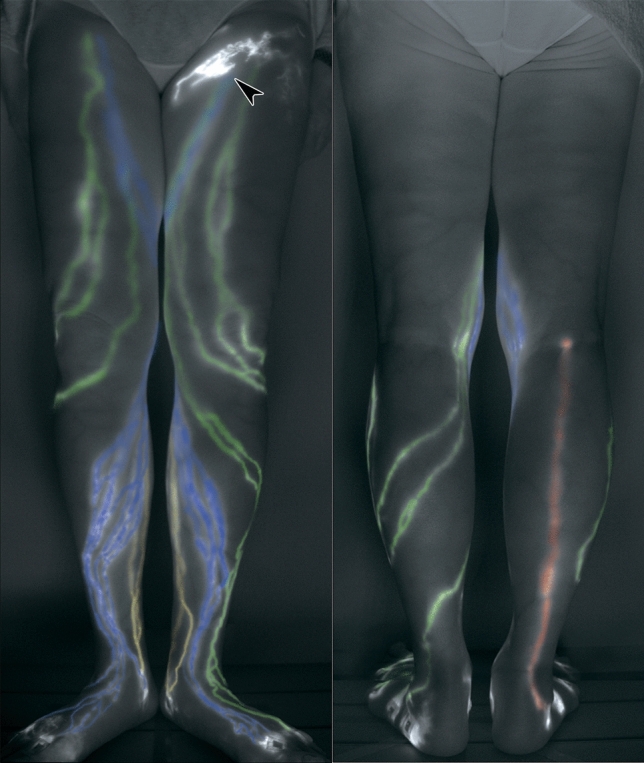
Changes in the lymphatics in lymphedema in the lower limb, Indocyanine green lymphographic image. Indocyanine green lymphographic images show the four-colored lymphatic vessels and dermal back-flow (arrowhead): anteromedial (blue), anterolateral (green), posteromedial (yellow), and posterolateral (red). An image comprised two aspects: anterior (right), and posterior sides (left). The image of the right leg showing four lymphatic groups and no dermal back-flow (DB). Left leg with mild lymphedema showing single defects of posterolateral group, in addition to inguinal DB (arrowhead).

**Figure 3:**
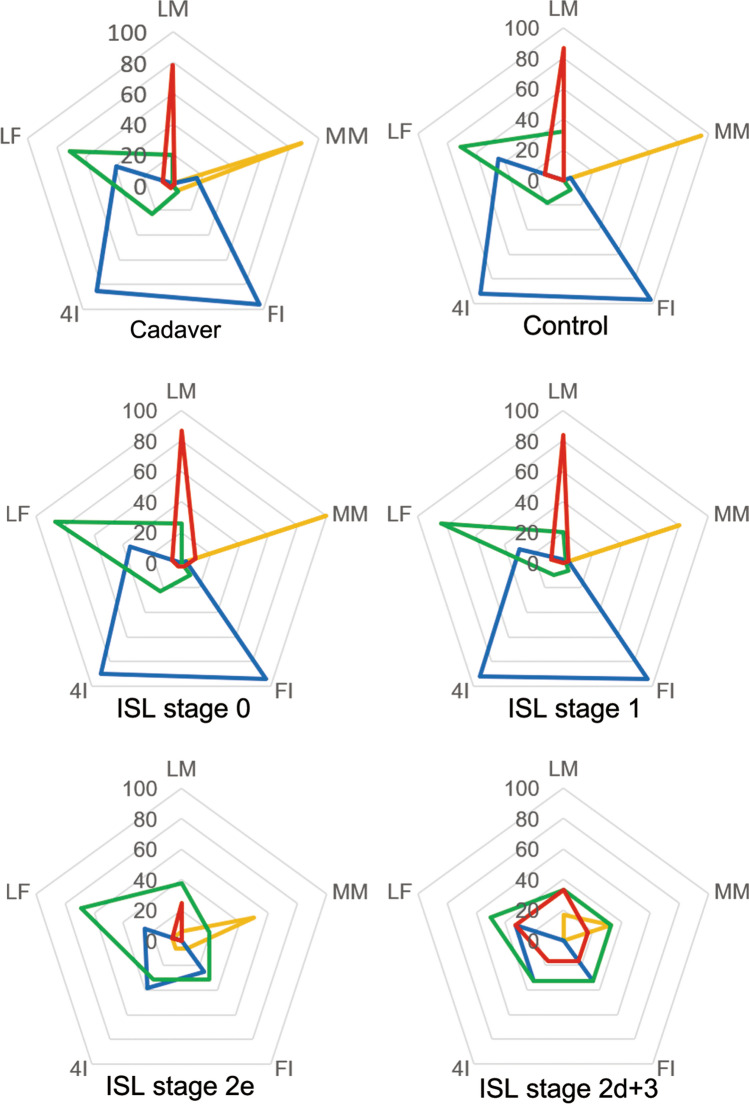
The relationship between injection sites and the lymphatic vessel groups: radar chart. The radar chart showed the percentage of visualization in the lymphatic pathways at each injection site. The number of axes shows the percentage of the visualization of each lymphatic pathway. The color of the radar line denote the four lymphatic pathways: anteromedial (blue), anterolateral (green), posteromedial (yellow), and posterolateral (red). In the controls, the rate of existence of the three lymphatic groups other than the anterolateral group exceeded 90%, and the use of four injection sites, other than the fourth interdigital space, made it possible to visualize the four lymphatic groups steadily. The mild lymphedema group (International Society of Lymphology [ISL] stage 0 + 1) had few defects in the lymphatic groups, and in ISL stage > 2, the defect rate in all lymphatic groups increased rapidly. The anterolateral group tended to be restricted to stage 3. *MM* medial malleolus, *FI* first interdigit space, *4I* 4th interdigit space, *LF* lateral of foot, *LM* lateral malleolus.

We checked for the existence of DB and colored the entire courses of the four lymphatic vessel groups (posteromedial: PM, posterolateral: PL, anteromedial: AM, and anterolateral: AL) from the injection sites to the lymph nodes according to the anatomical classification in the ICG fluorescent lymphography images (Fig. 2). When compared with our previous cadaveric data, the relationship between the injection sites and lymphatic groups was similar in the control group, but the rate of visualization for each of the injection sites was higher than that obtained in the cadaveric study. With the progress in the ISL stage and the decreased visualization of the lymphatic vessel groups, the territory of the remaining lymphatic groups spread and overlapped with each other (Fig. 3).

In the control group, DB was not observed, but in the lymphedema group, appearance of DB increased with progression in the ISL stage. Stages beyond stage 2 showed a sharp increase in the existence of DB, while all lower limbs in stage 3 had DB (Table 2). It was not possible to determine from which lymphatic vessel groups the DB appeared, because these lymphatic vessel groups are closely located. In the control limbs, there were no defects in the anteromedial and posteromedial groups, but the other two groups had a few defects: 21% (9/42) in the anterolateral and 4.8% (2/42) in the posterolateral groups. In the lymphedema limbs, with progress in the ISL stages, the rates of defects occurring in the three lymphatic groups, other than the anterolateral group, increased and approximately half of the lymphatic groups were defective in limbs with ISL stage 3. In the severe stages (stage 2d and stage 3), only the anterolateral group with 25% (6/24) was not defective compared to the other three groups (50% (12/24) in PM, 71% (17/24) in PL, and 58% (14/24) in AM) (Table 2).

**Table 2:** The relation between lymphedema severity, the DB and the defects of lymphatic vessel group.

		Lymphatic vessel group defect							
	Dermal back-flow	Posteromedial		Posterolateral		Anteromedial		Anterolateral	
	+ (n = 102) − (n = 104)	+ (n = 26)	− (n = 180)	+ (n = 37)	− (n = 169)	+ (n = 20)	− (n = 186)	+ (n = 30)	− (176)
Control (n = 42)	+ (n = 0)	0	0	0	0	0	0	0	0
	− (n = 42)	0	42	2	40	0	42	9	33
Stage 0 (n = 46)	+ (n = 12)	2	10	0	12	1	11	4	8
	− (n = 34)	0	34	5	29	0	34	3	31
Stage 1 (n = 35)	+ (n = 20)	1	19	2	18	2	18	2	18
	− (n = 15)	0	15	2	13	0	15	0	15
Stage 2e (n = 58)	+ (n = 46)	10	36	8	38	3	43	2	44
	− (n = 12)	1	11	1	11	0	12	4	8
Stage 2d+3 (n = 25)	+ (n = 24)	12	12	17	7	14	10	6	18
	− (n = 1)	0	1	0	1	0	1	0	1

**Figure 4:**
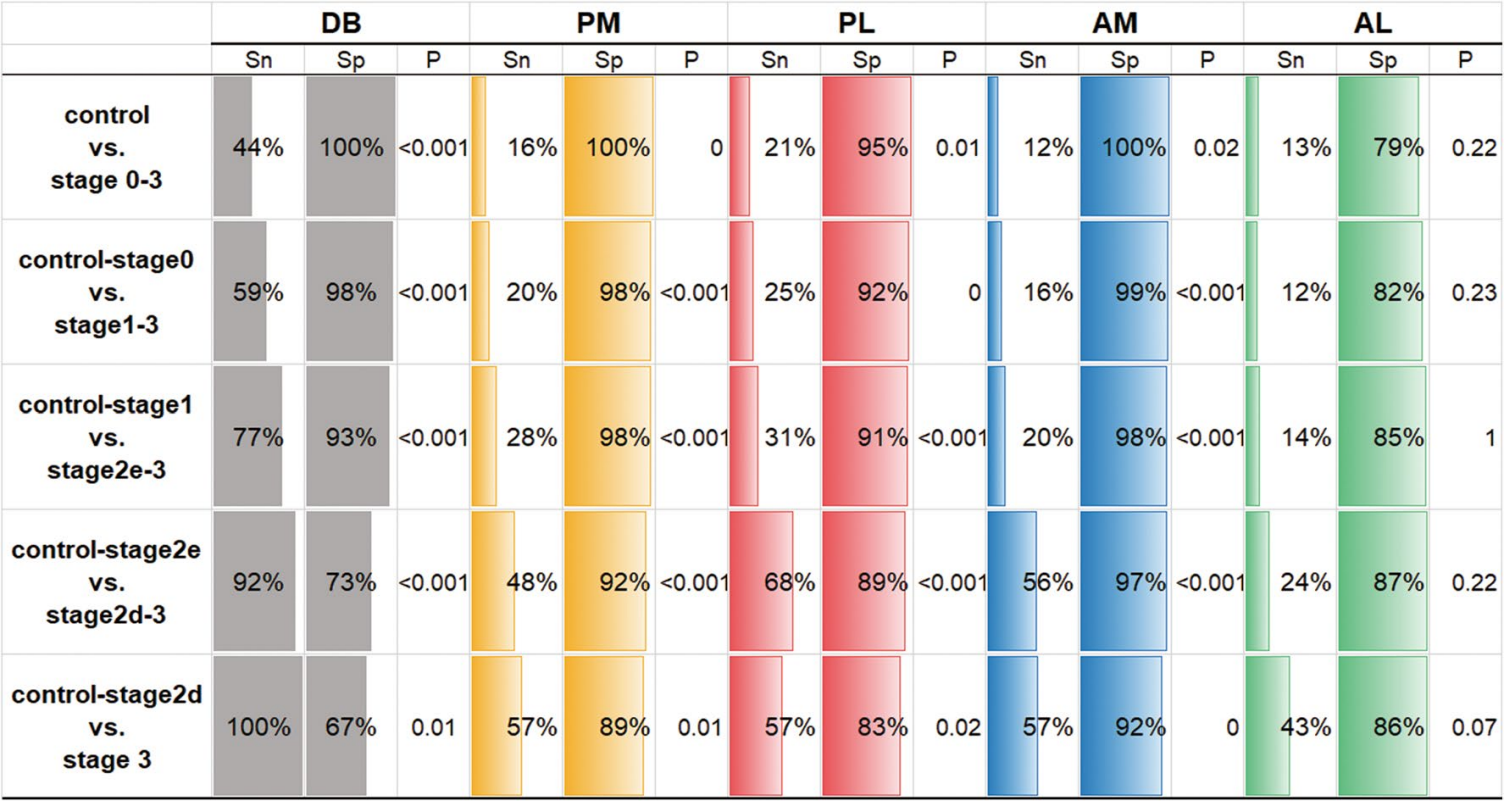
Sensitivity and specificity of dermal back-flow and defects in lymphatic groups for the diagnosis of lymphedema. Except for the anterolateral group, dermal back-flow (DB) and defects in the three lymphatic groups (anterolateral, posteromedial, and posterolateral) showed a statistically significant difference in distinguishing all stages. Although DB had low sensitivity but high specificity to detect lymphedema (control vs. International Society of Lymphology [ISL] stages 0‒3), it had high sensitivity but low specificity to judge severity (control–stage 2e vs. stage 2d‒3, and control–stage 2d vs. stage 3). Although each of three lymphatic group defects had low sensitivity for diagnosis and judgment of severity, they retained a high specificity for all stages. *Sn* sensitivity, *Sp* specificity.

The sensitivity and specificity of diagnosis and severity judgment using DB existence or defects in each lymphatic group as the predictor variable are shown in Fig. 4. With the exception of the defects in the anterolateral group, DB and defects in the other three lymphatic groups showed significant differences in distinguishing all stages (*p-*values shown in Fig. 4). Although DB had low sensitivity, but high specificity to detect lymphedema (control vs. stage 0‒3), it had high sensitivity but low specificity to judge whether lymphedema was severe (control‒stage 2e vs. stage 2d‒stage 3, and control‒stage 2d vs. stage 3). Although defects in the lymphatic groups had low sensitivity for distinguishing all stages, they retained specificity for distinguishing all stages.

**Figure 5:**
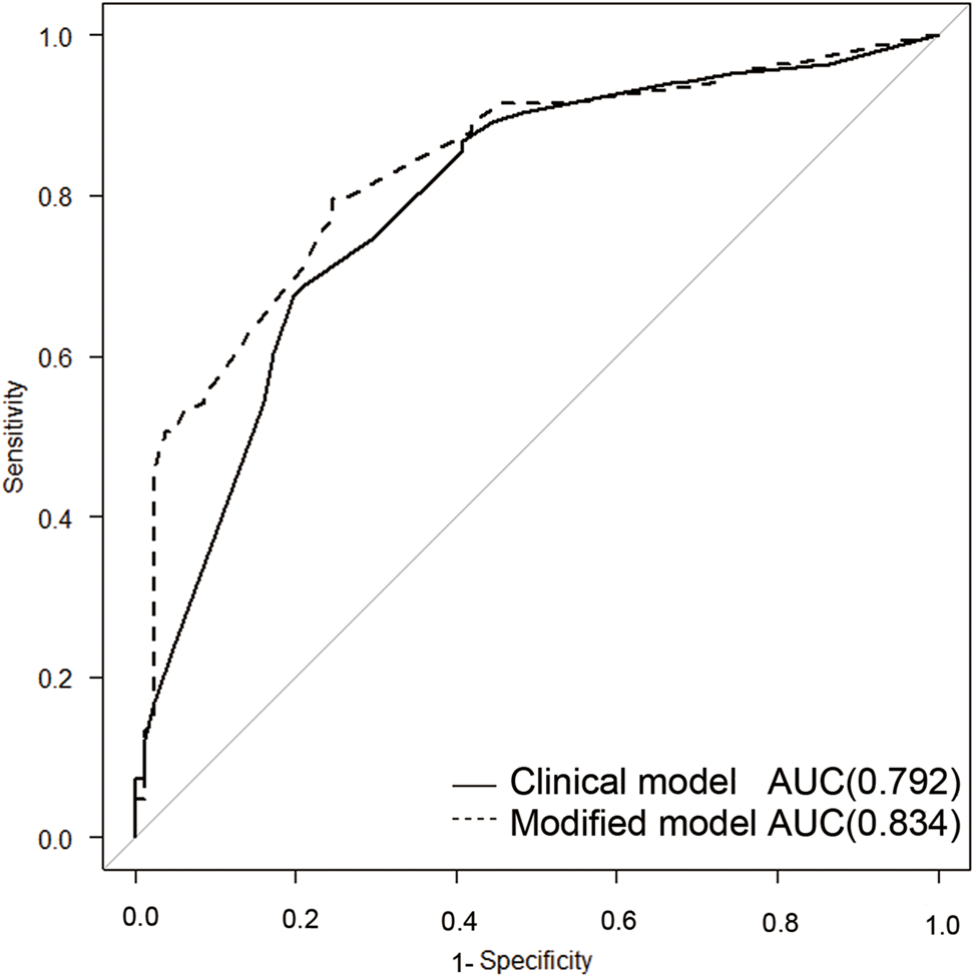
Receiver operating characteristic curves of the clinical model and the modified model. The area under the curve (AUC) of the modified model that included defects in the posteromedial and posterolateral lymphatic groups in addition to clinical risk factors was significantly higher than that of the clinical model that included only dermal back-flow and clinical risk factors.

The characteristics of patients with lymphedema included in the study are shown in Table 3, stratified by the existence of DB or defects in each of the lymphatic groups. Only age over 80 years with a defect in the posteromedial group and chemotherapy with DB showed statistically significant differences. Table 4 summarizes the characteristics that were evaluated separately for independent association with the severity of lower limb lymphedema. All individual characteristics with a *p*-value < 0.2 were then introduced into the multivariable model. DB, defects in the posteromedial and posterolateral groups, age over 80 years, and sides were identified to be significantly associated with the severity of lymphedema in the multivariable model. This new (modified) severity diagnosis model (AUC: 0.834) that implemented defects in the lymphatic groups had a more accurate prediction ability than the clinical model (AUC: 0.792) and was statistically significant (*p*  =  0.029) (Fig. 5). Lymphedema of lower limbs with double defect (PM and PL) was significantly more severe (*p* = 0.0247) than that with a single defect (PM or PL) (Table 5).

**Table 3:** Baseline characteristic for logistic regression analysis.

	Dermal back-flow			Defect of lymphatic vessel groups											
				Anteromedial			Anterolateral			Posteromedial			Posterolateral		
	+ (102)	− (62)	P-value	+ (20)	− (144)	P-value	+ (21)	− (143)	P-value	+ (26)	− (138)	p-value	+ (35)	− (129)	P-Value
**Clinical characteristics**															
Age over 80 years, n (%)	11 (10.8)	3 (2.9)	0.254	4 (20)	10 (6.9)	0.072	3 (14.3)	11 (7.7)	0.393	5 (19.2)	9 (6.5)	0.049	4 (11.4)	10 (7.8)	0.5
Female sex, n (%)	98 (96.1)	56 (90.3)	0.180	20 (100)	134 (93.1)	0.612	20 (95.2)	134 (93.7)	1	25 (96.2)	129 (93.5)	1	34 (97.1)	120 (93)	0.691
Body mass index kg/m^2^ over 25, n (%)	14 (13.7)	16 (25.8)	0.062	4 (20.0)	26 (18.1)	0.765	3 (14.3)	27 (18.9)	0.768	4 (15.4)	26 (18.8)	0.789	3 (8.6)	27 (20.9)	0.137
Right, n (%)	52 (51)	29 (46.8)	0.180	12 (60.0)	69 (47.9)	0.348	10 (47.6)	71 (49.7)	1	13 (50.0)	70 (50.7)	0.833	17 (48.6)	64 (49.6)	1
**Invasions for lymphatics**															
Lymph node dissection+, n (%)	92 (90.2)	49 (79.0)	0.063	19 (95.0)	122 (84.7)	0.313	21 (100)	120 (83.9)	1	24 (92.3)	117 (84.8)	0.537	33 (94.3)	108 (83.7)	0.168
Chemotherapy+, n (%)	53 (52.0)	43 (69.4)	0.034	10 (50)	86 (59.7)	0.471	10 (47.6)	86 (60.1)	0.344	12 (46.2)	84 (60.9)	0.195	15 (42.9)	81 (62.8)	0.052
Radiotherapy+, n (%)	31 (30.4)	12 (19.4)	0.144	8 (40)	35 (24.3)	0.174	5 (23.8)	38 (26.6)	1	11 (42.3)	32 (23.2)	0.053	9 (25.7)	34 (26.4)	1

**Table 4:** Univariable and multivariable predictors of lymphedema severity.

Variable	Univariable			Multivariable		
	OR	95% CI	p-value	OR	95% CI	p-value
Dermal back-flow	8.3	3.93–17.3	<0.001	6.42	2.78–14.8	<0.001
Anteromedial	6.7	1.88– 23.9	0.0033	0.787	0.16–3.89	0.77
Anterolateral	1.35	0.54–3.41	0.52			
Posteromedial	9.97	2.86–34.8	0.00031	4.68	1.05–20.8	0.043
Posterolateral	3.65	1.59–8.4	0.0024	3.50	1.15–10.7	0.028
Age over 80 years	6.68	1.45–30.8	0.015	6.77	1.31–35.0	0.022
Radiotherapy	1.96	0.96–4.00	0.065	1.88	0.76–4.64	0.17
Chemotherapy	0.629	0.34–1.18	0.15	1.10	0.49–2.47	0.82
Side	1.8	0.97–3.35	0.062	2.32	1.08–4.99	0.030
BMI over 25 kg/m^2^	1.14	0.52–2.52	0.74			
Lymph node dissection	1.14	0.47–2.75	0.77

**Table 5:** The relation between single or double defect and lymphedema severity.

	ISL lymphedema stage		
	0-2 early	2 delay-3	Total
Single defect (PM or PL)	24	11	35
Double defect (PL and PM )	4	9	13
Total	28	20	48

**Figure 6:**
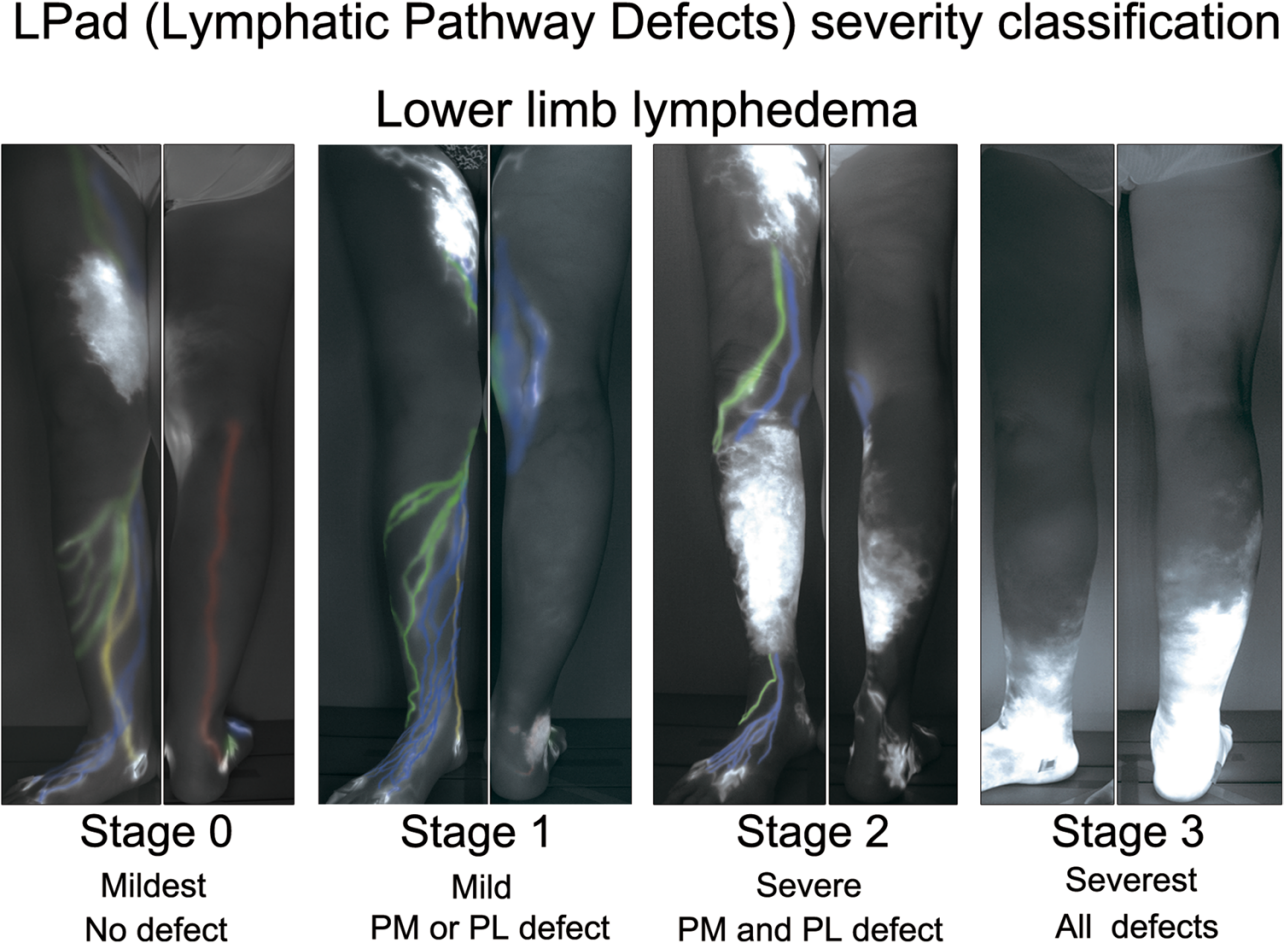
LPad (lymphatic pathways defects) severity classification of lower limb lymphedema. Indocyanine green lymphographic images of four lymphedema legs show the four-colored lymphatic vessel groups and dermal back-flow: anteromedial (blue), anterolateral (green), posteromedial (yellow), and posterolateral (red). Double defects of the posteromedial and posterolateral groups were more severe than a single defect of these two groups. Defects of all lymphatic groups, including the anteromedial and anterolateral groups, were more severe than double defects; previous reports have shown that in the terminal stage of leg lymphedema, all lymphatic collecting vessels in the legs are lost.

Based on the lymphatic vessel group defects, a new lymphedema severity classification (Lymphatic Pathway Defects severity classification: LPad severity classification) was created. Lymphedema of the leg without lymphatic group defect was the mildest stage, single defect in the posteromedial or posterolateral group indicated an increase in severity, and double defects in these groups indicated even greater severity (Fig. 6).

## Discussion

This study verified and confirmed the anatomical accuracy of the new injection technique for lower limb lymphography, which was modified based on anatomical studies. Precise information regarding the entire lymphatic anatomy of edematous legs would elucidate information regarding affected lymphatic pathways and target the lymph nodes for repair by surgical techniques or manual lymphatic drainage. Using a multivariable logistic regression model adjusted for risk factors of lymphedema severity, we demonstrated the clinical significance of defects in each lymphatic vessel group in lymphedema. DB is a sensitive and specific finding of lymphedema; however, it is unable to completely judge the severity. In contrast, defects in the lymphatic groups were useful in judging the severity of lymphedema specifically. Therefore, in addition to identifying DB, considering defects in the lymphatic vessel groups, especially the posteromedial and posterolateral groups, could improve the judgment of lymphedema severity. Finally, this information gave rise to a new lower limb lymphedema severity classification based on the defects in the lymphatic pathways. The LPad severity classification would be superior to the consensus classifications as our classification was based on the mechanism of the pathogenesis of lymphatic dysfunction while the previous classifications were based on the phenotypes (such as symptoms) as a result of the lymphatic dysfunction.

### Site of the lymphatic defect and impact on lymphedema severity

This study confirmed that anatomical changes in lymphatics did not only stage lymphedema, lymphatic changes mainly manifested as DB; however, as lymphedema progressed, the number of defects in the lymphatic groups increased. While defects in the posteromedial and posterolateral groups were strongly related to severity independently, those in the anteromedial and anterolateral groups showed no relationships with severity. An anatomical study demonstrated that only the anterolateral group had a possibility (over 40%) of connecting with the lower abdomen lymphatic territory^26^; thus, in severe lymphedema, the anterolateral group acted as a compensatory collateral. In addition, this could also be because both the anteromedial and anterolateral groups run superficially in the subcutaneous layer and along the branches of the great saphenous vein, while the posteromedial and posterolateral groups run on the deep fascia and along the main trunk of the great saphenous vein and the lesser saphenous vein^26^. Thus, it seems that the anteromedial and anterolateral groups are minor lymphatic pathways anatomically, and in a leg with lymphedema, they might function as collateral pathways in compensation for two major pathways (posteromedial and posterolateral groups) damaged antecedently. However, it is well known that in most cases of severe lymphedema of the leg, all the lymphatic vessels, including the collateral pathways, are lost^27,28^. Therefore, in the lymphedema severity classification based on defects of the lymphatic vessel groups, the most severe stage would involve defects of all lymphatic vessel groups, including the anteromedial and anterolateral groups (Fig. 6).

### Impact on lymphatic imaging methodology

Although previous injection methods of tracer or other contrast materials for lymphography involved a single point in the interdigital space, we showed that injecting only at the interdigital space can visualize only the anteromedial group, and visualizing all four lymphatic groups requires the use of at least three additional injection sites outside the interdigital space. It is not clear whether visualizing all four lymphatic groups can improve DB detection; however, it does enhance judgment of the severity of lymphedema. Although defects in the two major lymphatic groups were related to the severity of lymphedema, defects in the two minor groups did not show a statistically significant effect on the severity. Thus, evaluation of the two minor groups is not useful for the judgment of lymphedema severity but is important for confirming healthy collaterals to the region; this information could be useful for planning manual lymphatic drainage and lymphatic surgeries, such as LVA. Therefore, for an accurate examination, it is imperative not only to evaluate the two major lymphatic groups but also the two minor lymphatic pathways.

### Relationship between lymphatic group defects and lymphedema treatment

For treatments aimed at improving lymphatic drainage, such as manual lymphatic drainage, LVA, and LNT, it is essential to understand changes in lymphatic pathways. The current study helps to define normal lymphatic mapping, and our injection technique can definitively show the remaining lymphatic pathways and functioning lymph nodes for use in treatments in patients with lymphedema.

This study had certain limitation. First, it had a retrospective design. Second, there was a sex-bias for females, and male participants included had only the severe type of lymphedema. Thus, the obtained data could not demonstrate the effect of sex on the severity, and the analyses could not be adjusted for sex. Moreover, as ICG lymphography can visualize only lymphatic vessels, but not lymph nodes clearly, this study strongly suggested, but could not prove directly that lymphedema involves damage of the lymph nodes corresponding to each lymphatic vessel group.

The limitations of this study include the lack of information regarding the depth due to the ICG fluorescence lymphography being observed from the surface. Accordingly, the addition of other techniques for evaluating the information regarding the depth will be needed.

## Methods

Between August 1, 2017 and July 21, 2019, 84 consecutive patients (164 lower limbs) who underwent ICG fluorescent lymphography at Kagawa Prefectural Hospital and Okayama University Hospital were registered as the lymphedema group. Twenty-one participants (42 lower limbs) who underwent ICG fluorescent lymphography before gynecologic cancer treatments in other clinical trials (no. 396) at Kagawa Prefectural Hospital were registered as the control group. Experiments were conducted in accordance with the guidelines of the Declaration of Helsinki Principles and informed consent was obtained from all participants. The lymphedema group was defined as participants who underwent lymph node dissection, radiotherapy to pelvic or inguinal lymph nodes, or taxane chemotherapy. Patients who were suspected of venous dysfunction by venous sonography, dysthyroidism, or heart failure based on blood test results were excluded from this study.

ICG lymphography was performed by a single plastic surgeon (A.S.), who had experience with over 1000 cases of ICG lymphography over a 10-year period, and the data were rechecked by another plastic surgeon who had more than 5 years’ experience as a lymphedema specialist.

As a lymphedema severity scale, the International Society of Lymphology (ISL) staging system was used, and the stages were determined by two nurses who each had 5 years’ experience as lymphedema therapists. All data were abstracted by A.S. from medical records and were later rechecked by a member of the research team to confirm accuracy.

The Ethics Committees of Kagawa Prefectural Hospital (no. 860) and Okayama University Hospital (K1909-205) approved this investigation.

### ICG fluorescent lymphography

ICG 25 mg (Diagnogreen^®^; Daiichi Sankyo Co., Ltd., Tokyo, Japan) was diluted with 10 ml of pure water. Based on past anatomical studies, five injection sites were marked around the foot along the border between the dorsum and the planta; below the medial malleolus, below the lateral malleolus, in the first interdigital space, fourth interdigital space, and at the midpoint of the straight line that connects the head of the fifth metatarsal bone and the lateral malleolus (Fig. 1). ICG solution (0.2 ml) was subcutaneously injected using a 1-ml syringe with a 30-G needle. Pain control was not performed during the ICG injection. Immediately following the injections at all sites, gentle hand massage was performed at each injection site and thereafter to the lower limb. The lymphatic vessels were identified using a near-infrared camera system (pde-neo^®^; Hamamatsu Photonics K.K., Shizuoka, Japan) consecutively for 5 min and then, finally checked for the appearance of DB following a 15-min walk^29^. The excitation light was kept at the maximum position, while the contrast and gain were kept fixed at the central position throughout the examinations. After 15-min walk, sequential still images were taken using a self-made camera with slider system, and the images were montaged with an imaging software (Image Composite Editor, Microsoft Corporation, Redmond, WA). The self-made camera consisted of a near-infrared camera (UI-3240CP-NIR-GL ReV.2; Imaging Development Systems GmbH, Obersulm, Germany), long pass filter (IR long pass filter 9023521; Laser Create Corp., Tokyo, Japan), machine vision lens (LM8HC-SW; Kowa Optronics Co., Ltd., Tokyo, Japan), and near-infrared light emitting diodes (750-nm High Power TOP LED SMBB750-1100-05; Ushio Inc, Tokyo, Japan). The power of excitation light was kept at 3–4 mW/cm^2^ at 50 cm from front of lens. The settings of near-infrared camera were as follows: frame rate, five frames/s; gain, 60 dB; and gamma, off.

The lymphatic vessels in the lower limb were classified into four lymphatic groups: anteromedial, anterolateral, posteromedial, and posterolateral (assessed by A.S.), as detailed in a previous report^25^. DB and defects in each lymphatic group were recorded. DB patterns (splash, star dust, and diffuse) were not distinguished in this study.

### Data analysis

Data are summarized using standard descriptive statistics. Patient characteristics were compared between groups (control vs. lymphedema) using Fisher’s exact test for categorical variables.

To consider the sensitivity and specificity for diagnosis of lymphedema and severity judgment, we used the existence of DB and defects in each lymphatic group as predictor variables. The control vs. lymphedema groups were considered as the objective variables for diagnosis of lymphedema and control–stage 0 vs. stage 1‒stage 3, control–stage 1 vs. stage 2‒stage 3, control–stage 2 early (e) vs. stage 2 delay (d)‒stage 3, and control–stage 2 vs. stage 3 were considered as the objective variables for severity judgments.

For patients with lymphedema, we used univariable logistic regression models to evaluate the individual risk factors associated with lymphedema severity in previous studies^6–13^. Sex was excluded from the risk factors because there was a sex-bias for female participants, and the included male participants had only severe type of lymphedema in this study. Finally, age over 80 years, body mass index (BMI) over 25 kg/m^2^, right or left side, lymph node dissection, radiotherapy, and taxane chemotherapy were selected as risk factors. In the lymphedema patient group, stages 0 and 1, which involve subtle lymphatic damage, were classified into the mild lymphedema group, while stages 2 and 3, which involve overt lymphedema, were classified into the severe lymphedema group. A multivariable logistic regression model was then established using factors with *p*-value < 0.2 in univariable analysis. Associations were summarized by calculating odds ratios (ORs) and corresponding 95% confidence intervals (CIs) from the model parameter estimates.

To consider the power of the new severity diagnosis, the predicted probability for both a clinical model and a modified model were calculated using a generalized linear model. The clinical model used DB and other risk factors that had *p* < 0.2 in univariable analysis (age over 80, radiotherapy, chemotherapy, and side), and the modified model used the factors in the clinical model and lymphatic group defects with plus p < 0.2. Receiver operating characteristic curves were drawn according to the predicted probabilities, and the areas under the curve (AUCs) were calculated and compared using the Delonghi test.

To consider the difference between double defect and single defect of posterolateral and posteromedial group, the severity distributions (stage 0-stage 2 early vs. stage 2 delay-stage 3) were compared using Fisher's exact test.

A *p* < 0.05 was considered statistically significant. Statistical analysis was performed using the EZR soft package ver.2.6-1 based on R software (https://www.r-project.org/)^30^.
